# Convergence of Intelligent Data Acquisition and Advanced Computing Systems

**DOI:** 10.3390/s21072262

**Published:** 2021-03-24

**Authors:** Grigore Stamatescu, Anatoliy Sachenko, Dan Popescu

**Affiliations:** 1 Department of Automation and Industrial Informatics, University Politehnica of Bucharest, 313 Splaiul Independentei, 060042 Bucharest, Romania; dan.popescu@upb.ro; 2 Department of Informatics, Kazimierz Pulaski University of Technology and Humanities in Radom, ul. Malczewskiego 29, 26-600 Radom, Poland; as@wunu.edu.ua; 3 Research Institute for Intelligent Computer Systems, West Ukrainian National University, 3 Peremoga Square, 46020 Ternopil, Ukraine

This editorial article briefly outlines the objectives and achieved goals of the Special Issue on “Convergence of Intelligent Data Acquisition and Advanced Computing Systems” running between September 2019 and September 2020 in the *Sensors* journal. The special issue welcomed submissions as extended versions of conference articles published in the proceedings of the “10th IEEE International Conference on Intelligent Data Acquisition and Advanced Computing Systems: Technology and Applications (IDAACS’2019)” allowing the authors to expand on their theoretical and experimental contributions. These were complemented with external submissions from interested researchers working in this timely area. We highlight the main contributions of the published articles, grouped by key topics: intelligent data acquisition, learning methods and optimisation for data processing and advanced computing systems to support data processing.

As modern data acquisition solutions increasingly integrate on-board computing, in the case of intelligent sensors, or in-network computing, in the case of distributed systems and sensor networks, a need has been observed to provide a joint view on these previously divergent topics. We define the convergence area of intelligent data acquisition and advanced computing systems at the interface of theory and applications for industrial manufacturing, scientific computing, precision agriculture, energy systems such as smart building and smart city systems. This bridges specific topics for instrumentation such as accuracy, sampling, time synchronisation, sensor selection and calibration with algorithm design, statistical and machine learning, computational efficiency, reconfigurable computing that support the conventional engineering tasks. Computing-as-a-service is another relevant approach that can be leveraged to improve measurements and instrumentation in the future.

Potential topics of the special issue that were initially defined were mapped onto the core areas and special streams of the IDAACS’2019 conference, and included but were not limited to: advanced instrumentation and data acquisition systems, advanced mathematical methods for data acquisition and computing systems, computational intelligence for instrumentation and data acquisition systems, data analysis and modeling, intelligent distributed systems and remote control, intelligent information systems, data mining, and ontology, Internet of Things, pattern recognition, digital image, and signal processing, intelligent instrumentation and data acquisition systems in advanced manufacturing for Industry 4, intelligent robotics and sensors, machine learning, application for smart buildings and smart cities.

The special issue received 25 submissions which, after a thorough and competitive review process, resulted in nine published articles. The accepted articles have been co-authored by 41 authors in total from 14 countries, with good geographical diversity. Performing an overview of the accepted articles, we group them into three main categories focused on data acquisition, modern data processing algorithms and computing architectures that support data processing.

Intelligent data acquisiton and data collection platforms: This section highlights three articles that focus on data acqusition from sensor and sensor platforms in electrical vehicles, precision agriculture and educational robotics applications.

In [1], the authors provide an experimental evaluation of time delay estimation methods for current measurements in electrical vehicle powertrains. These include linear regression (LR), variance minimization (VM) and adaptive filters (AF). The methods are benchmarked in terms of Root Mean Squared Error (RMSE) and average run-time on data collected from realistic driving profiles. Given its efficiency, the VM method is recommended for this application considering noise resistance and computational efficiency. The study is highly relevant also considering the computational constraints of distributed electronic control units (ECU) in modern automobiles.

Ref. [2] presents a system that combines scalar, ground level measurements, from sensor networks with aerial robotic platforms (UAVs) as data collection and communication relay infrastructure. Main innovation lays in the in network data aggregation by consensus in order to reduce the need for data transmissions. The flight path of the UAV is optimized using spline functions in order to maximize the flight time and data gathering in a given area from the cluster heads. A symbolic aggregation approximation (SAX) method encodes the raw sensor measurements into text strings for each of the monitored parameters in the precision agriculture application. Results quantify both the data reduction as well as the UAV performance improvement using the trajectory control scheme.

A sensor-intensive robotic platform for education is introduced by [3]. A thorough review of existing educational robotic platforms is carried out alongside the argumentation for the Robotic Operating System (ROS) as framework of choice for the underlying implementation and new developments. The system is built around a Raspberry Pi embedded development board with dedicated function specific sensors such as ultrasonic, wheel encoders, LIDAR, and camera. It can be controlled over WiFi communication interface using a PC or a mobile phone. Its performance is experimentally validated through a series of tests as well as in qualitative studies using various groups of students.

Learning from data and optimization: This section focuses on four articles that cover statistical and machine learning and optimization techniques that make use of collected sensor data together with advanced computing algorithms for implementation.

In [4], a detailed study is provided for smart building system identification combining both classical, as non-linear autoregressive (NARX) models, and data-driven machine learning based approaches, as multi-layer perceptrons (MLP). The goal is to achieve an accurate model of building thermal dynamics for control using collected data under various conditions. Data are colected through existing building automation system (BAS) infrastructure with wireless components for sensing, room temperature sensors, and control, motor valves for the heating elements. The study is carried out on a real building from University Paris-Est Creteil (UPEC). The main results show good performance in terms of Mean Squared Error (MSE) and Mean Absolute Error (MAE) of less than 0.2C compared to the ground truth.

Ref. [5] introduces an improved deep learning method for evaluation and classification of road quality based on the U-Net deep autoencoder. The main innovation stems from the addition of residual connections, atrous spatial pyramid pooling and attention gates to increase performance. Evaluation is performed on multiple reference benchmarking datasets: CrackForest, Crack500, GAPs384. The Dice score is used for comparing various architectures and parametrisation options of the deep learning architectures with a robust improvement of the proposed ResU-NET + ASPP + AG network over the baseline specfication. Testing has been done on dedicated graphical processing units, on each dataset, while mixed dataset training did not yield consistent results.

A mixed integer linear programming optimization problem for smart parking EV charging is formulated by [6]. The goals are two-fold: to maximize the parking lot revenue through accommodating charging EVs as efficiently as possible and minimizing the cost of power consumption through participation in the utility-level demand response (DR) program. The study is validated in simulation using predefined schedules and model EV charging characteristics. A simplified convex relaxation technique is introduced to assure feasibility of the optimization problem. The solution is compared against a standard variable charging power approach and shows consistent improvements in terms of power consumption cost and percentage savings.

The authors of [7] propose a new scheme for data classification by combining sparse auto-encoders (SAE) with data post processing using a nature-inspired particle swarm optimization (PSO) algorithm. The post processing layer improves the classification performance of the deep neural network through a parameter optimized linear model. Labeled datasets used for practical evaluation stem from the medical field and include epileptic seizures, SPECTF and cardiac arrhythmias while experimenting with multiple parameters of both the neural network and the PSO algorithm. The adjustment layer improves the performance of the models as illustrated through other documented studies from the literature, achieving for example a 99.27% accuracy on the cardiac arrhythmia dataset.

Advanced computing systems for data processing: This section includes two articles that present improvements to data processing through optimized architectures for solving differential equations and reconfigurable computing with FPGAs.

Ref. [8] approach the problem of increasing performance of solving ordinary differential equations (ODE) on multi-core embedded systems which can describe the system model of certain physical phenomena. The authors introduce an adaptive algorithm, PAMCL, based on the Adams-Moulton and Parareal methods and provide a comparison with existing approaches. Implementation-wise OpenCL platform is used with optimized solvers for both CPU and GPU systems. Quantitative results are reported that include the CPU run time, GPU speed-up and memory footprints of the reference algorithms. The method shows good results and achieves full convergence to the exact solutions. A potential extension of the PAMCL method for partial derivative equations is described.

Power-oriented monitoring of clock signals in FPGA systems is described by [9]. The argumentation of the work lays in the need to reduce power consumption and checkability of reconfigurable computing platforms. The study includes two types of power-monitoring: detection of synchronization failures and dissipation of power using temperature and current sensors. Experiments are carried our on typical computing tasks, e.g., digital filter implementations, using standardized tools for monitoring and data collection. Thermal and power dissipation data is associated with fault conditions in the synchronization. Such improvements to the evaluation of FPGA systems are highly relevant for critical, high reliability, applications.

## References

[1] Pfeiffer J., Wu X., Ayadi A. (2020). Evaluation of Three Different Approaches for Automated Time Delay Estimation for Distributed Sensor Systems of Electric Vehicles. Sensors.

[2] Popescu D., Stoican F., Stamatescu G., Ichim L., Dragana C. (2020). Advanced UAV–WSN System for Intelligent Monitoring in Precision Agriculture. Sensors.

[3] Karalekas G., Vologiannidis S., Kalomiros J. (2020). EUROPA: A Case Study for Teaching Sensors, Data Acquisition and Robotics via a ROS-Based Educational Robot. Sensors.

[4] Sadeghian Broujeny R., Madani K., Chebira A., Amarger V., Hurtard L. (2020). Data-Driven Living Spaces’ Heating Dynamics Modeling in Smart Buildings using Machine Learning-Based Identification. Sensors.

[5] Augustauskas R., Lipnickas A. (2020). Improved Pixel-Level Pavement-Defect Segmentation Using a Deep Autoencoder. Sensors.

[6] Jawad M., Qureshi M.B., Ali S.M., Shabbir N., Khan M.U.S., Aloraini A., Nawaz R. (2020). A Cost-Effective Electric Vehicle Intelligent Charge Scheduling Method for Commercial Smart Parking Lots Using a Simplified Convex Relaxation Technique. Sensors.

[7] Karim A.M., Kaya H., Güzel M.S., Tolun M.R., Çelebi F.V., Mishra A. (2020). A Novel Framework Using Deep Auto-Encoders Based Linear Model for Data Classification. Sensors.

[8] Tavakkoli V., Mohsenzadegan K., Chedjou J.C., Kyamakya K. (2020). Contribution to Speeding-Up the Solving of Nonlinear Ordinary Differential Equations on Parallel/Multi-Core Platforms for Sensing Systems. Sensors.

[9] Drozd O., Nowakowski G., Sachenko A., Antoniuk V., Kochan V., Drozd M. (2021). Power-Oriented Monitoring of Clock Signals in FPGA Systems for Critical Application. Sensors.

